# Development and evaluation of a psychological adjustment communication system for adolescents with polycystic ovary syndrome at a high risk of depression: A mixed-method study protocol

**DOI:** 10.3389/fpsyt.2022.937280

**Published:** 2022-11-15

**Authors:** Huiwen Tan, Li Gao, Yunmei Guo, Ying Liu, Rui Ding, Xin Yan, Xueting Wang, Yousha Wang, Lianhong Wang

**Affiliations:** ^1^Nursing Department, Affiliated Hospital of Zunyi Medical University, Zunyi, China; ^2^Nursing College, Zunyi Medical University, Zunyi, China; ^3^Department of Reproductive Medicine, Affiliated Hospital of Zunyi Medical University, Zunyi, China

**Keywords:** polycystic ovary syndrome, adolescents, psychological adjustment communication system, depression, mixed-method study

## Abstract

**Background:**

Depression is a serious psychological disorder that causes substantial psychological and physical suffering in adolescents, contributes to over 50% of suicide attempts, and affects the health status and psychosocial functioning of 25% of the adult population. The prevalence of polycystic ovary syndrome (PCOS) in adolescents is 5.6∼11.04%, and the prevalence of depression in adolescents with PCOS is as high as 50–60%. Depression seriously affects the rehabilitation and quality of life of adolescents with PCOS. In this study, we present a protocol for a mixed-method study to develop and evaluate the effectiveness of a psychological adjustment communication system for reducing the prevalence of depression among adolescents with PCOS who are at a high risk of depression in China. This study utilizes social support theory to develop a mobile phone-based intervention for adolescents with PCOS at a high risk of depression.

**Methods:**

This sequential exploratory mixed-method study consists of four consecutive phases. In the first phase, we will review the literature to understand the disease experience and needs of adolescents with PCOS to construct an initial psychological adjustment communication system. In the second phase, researchers will interview patients and their parents using purposeful sampling methods and semi-structured interviews to appropriately modify the psychological adjustment communication system. In the third phase, the Delphi method will be used to improve the psychological adjustment communication system. The fourth phase will employ a quantitative approach using a before-and-after design to measure the effectiveness of the system.

**Discussion:**

The results of this study will indicate the effectiveness of the psychological adjustment communication system in adolescents with PCOS who are at a high risk of depression.

**Registration number:**

ChiCTR2100050123.

## Introduction

Polycystic ovary syndrome (PCOS) is a common endocrine metabolic disorder whose causes are not yet known. The prevalence of PCOS among female adolescents is 5.6–11.04% (1), and the clinical manifestations of the disease include irregular menstruation, infertility, hyperandrogenemia, and ovarian polycystic degeneration (2). Typically, PCOS begins after puberty and persists throughout the entire reproductive cycle, including puberty, reproductive age, and the postmenopausal period. In addition to influencing reproductive function, it also increases the risk of type 2 diabetes, cardiovascular disease, and endometrial cancer (3). Simultaneously, PCOS is significantly related to the prevalence of mental illness. The prevalence of depression in adolescents with PCOS is approximately 50%, 2.4 times higher than that in normal girls of a similar age, and may be as high as 60% in obese patients (4, 5). As a serious mental illness, depression has negative physical, psychological, social, and behavioral effects and is also the most common cause of suicide, with more than 50% of adolescent suicides being related to depression (6–8). In addition, it interferes with the successful transition from adolescence to adulthood: nearly 25% of adults suffer from depression during adolescence, which seriously affects their health and psychosocial functioning as adults (9). Currently, there is no effective cure for PCOS; thus, self-management by patients is essential to improve the clinical symptoms and outcomes of the disease and is recognized by international guidelines as the first-line treatment for PCOS (10). However, a depressive state can negatively influence the treatment of PCOS, with some patients experiencing psychological stress and increased mental burden that leads to emotional stress (11) and an inability to engage in effective self-management (12) and affects the level of endocrine metabolism, limits the improvement in the original symptoms, and seriously affects the patients’ quality of life (13, 14).

For these reasons, depression in adolescents with PCOS has emerged as a serious problem with important adverse effects on health status, and is a health issue that must be addressed and taken into consideration during PCOS disease treatment. The PCOS Endocrinology Society’s clinical practice guidelines first recommended identifying depression in adolescents with PCOS in 2013 (15), while the 2020 international evidence-based guidelines for adolescents with PCOS emphasize the need to pay attention to identifying depression in adolescents with PCOS and providing timely intervention and treatment where necessary for those with depression (16). Due to the high prevalence of depression in adolescent PCOS patients, early detection of symptoms, identification of high-risk patients, and interventions to move the treatment window forward are critical to prevent, stop, and delay the progression of PCOS and improve patients’ outcomes (17, 18).

At present, the treatment of depression is relatively mature and consists mainly of a combination of drug therapy and psychological interventions. As a high-risk group for depression, PCOS patients do not meet the criteria for clinical drug treatment; therefore, blindly administering drug treatment may result in excessive treatment, but allowing the symptoms to develop without treatment may result in eventual development of depression (19). Psychological interventions have been widely used to study adolescent depression in high-risk groups. However, the effectiveness of these interventions is controversial because a review of the literature showed that cognitive-behavioral therapies, modified cognitive-behavioral therapies, and interpersonal therapy did not have a positive influence on reducing depression among adolescents (20). An additional systematic review demonstrated that cognitive behavior therapy-based psychotherapy for children and adolescents at a high risk for depression reduced depression prevalence by approximately 19%, but the duration of the therapy was only 12 months (21). Generally, psychological interventions include health promotion, preventive interventions, psychological counseling, and psychotherapy. Psychotherapy is generally aimed at those experiencing psychological disorders, whereas preventive interventions are aimed at high-risk groups, with measures intended to reduce risk factors and enhance protective factors to reduce the probability of developing a psychological disorder. Psychological treatment, rather than preventive interventions, have been shown to be the main approach for people at risk of depression. However, blind application of psychological treatment in high-risk groups may yield suboptimal results owing to the imprecise intervention measures as well as increase the psychological burden on adolescents by overtreating them. Furthermore, psychological treatment is significantly more expensive than preventive interventions in terms of both human and material resources, potentially resulting in wastage of medical resources. Consequently, the application of effective psychological interventions for groups at a high risk of depression is a topic that needs to be be addressed urgently.

The influence of stressors disrupts the balance among cognition, emotion, behavior, and subjective and objective environments after the perception of external stimuli (22). To restore the balance between subject and object, the body initiates psychological adjustment by regulating negative emotions and changing the psychological state, which includes both self-adjustment and external environmental adjustment (23). However, due to their immature cognitive and emotional regulation functions as well as their generally weak psychological resilience, adolescents show limited ability to cope with stressors independently, which is an important risk factor for depression. Thus, an effective social environment is an essential component of the external environment, and effective social support is an important protective factor that prevents the occurrence of depression.

The theory of social support originated in the 1960s from the concept of social circles, and was originally used to explore the impact of life stress on physical and mental health. With advancements in research, social support was introduced into the field of psychology, and its role in stress reduction was discussed (24, 25). Social support theory emphasizes the relationships among individuals’ use of environmental resources, their reactions to the social environment, and the interrelationship between people and various systems in the environment. The types of social support can be divided into three categories based on their sources: (1) helpful support, which assists individuals in reducing environmental stress; (2) emotional support, which offers encouragement and moral support; and (3) affirmative support, which confirms individuals’ abilities to cope with stress and their belief that stress may be overcome. Our earlier study on the experiences of women with PCOS revealed that these women require information and emotional support from medical staff, family members, and peers (26). Accordingly, this study is based on the theory of social support that considers the needs of patients’ disease experience, providing them with social support in terms of helpfulness, emotionality, and affirmation that will enable them to make a positive psychological adjustment, actively manage stress, and eventually improve their mental health.

**FIGURE 1:**
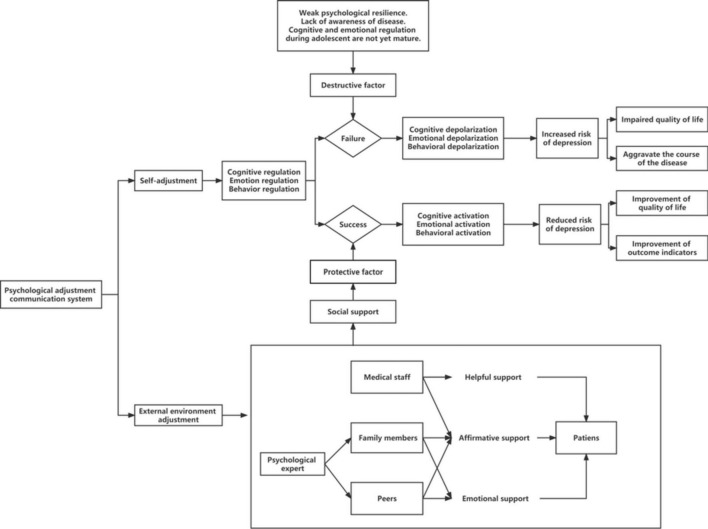
A conceptual framework for the psychological adjustment communication system based on social support theory.

The mobile network combines the advantages of mobile devices, such as cell phones, and the Internet. With recent advancements in mobile networks in China, interventional methods based on mobile networks have penetrated the medical services industry. In comparison with traditional face-to-face interventions, mobile network-based interventions offer the advantages of a wider audience, flexible timing, and low cost, and online parent-child communication significantly negatively predicts adolescent depression (27). According to the National Study on Internet Usage of Minors in 2020, there were 183 million Internet users among minors in China, and the Internet penetration rate among minors was 94.9%. Therefore, this study proposes the use of a social support-based psychological adjustment communication system using a mobile network for intervention in adolescents with PCOS at a high risk of depression. The conceptual framework of this study is illustrated in Figure 1

## Methods

Using an exploratory sequential mixed design, we will establish a social support theory framework based on helpfulness, emotionality, and affirmation, and position the roles of medical staff, peers, and family in these aspects of the system. The study will be conducted in four phases. The first phase will consist of a review of the related literature to understand the disease experience and needs of adolescents with PCOS, allowing the initial construction of a psychological adjustment communication system.

The second phase will involve qualitative interviews about the patient’s experience of psychological distress and the content of their needs in the helpful, emotional, and affirmative psychological support module, understanding the training needed for families to provide emotional psychological support to adolescents with PCOS at a high risk of depression, and modifying and improving the theoretical module of the psychological adjustment communication system.

The third phase will follow a quantitative approach implemented to modify and improve the psychological adjustment communication system, and score and guide the importance and maneuverability of the psychological adjustment communication system using the Delphi method.

**FIGURE 2:**
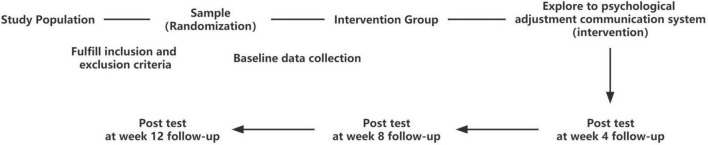
Consort diagram for the experimental study on the psychological adjustment communication system intervention’s effectiveness.

Phase 4 is a quantitative study to validate and evaluate the effectiveness and feasibility of the intervention and ascertain whether the desired outcomes have been achieved. Validation will be conducted on the basis of the objectives of (i) the usability of the psychological adjustment communication system and (ii) the effectiveness of the intervention program for adolescents with PCOS at a high depression risk. The effectiveness of the intervention will be assessed according to the set effect indicators between the baseline and follow-up at weeks 4, 8, and 12. Figure 2 shows the CONSORT diagram of the experimental study.

### Phase 1: Literature review

First, we will review a wide range of literature and materials using Johns Hopkins Evidence-Based Nursing Practice Theory to understand the disease experience and needs of adolescents with PCOS. The second step will involve developing a detailed search strategy and conducting a comprehensive literature review. We will search PubMed, ScienceDirect, Web of Science, PsycINFO, Cochrane Library, and other databases and guideline websites and evaluate them according to the Johns Hopkins Evidence Rating and Quality Criteria. Inclusion criteria will be defined on the basis of the PICOS question as follows: Population: adolescent PCOS patients aged 10–19 years; Interest of phenomena: the real experiences, feelings, and needs of PCOS patients after diagnosis; Context: the life experiences of PCOS patients during or after diagnosis and treatment; Study design: qualitative research, including phenomenology, rooted theory, ethnography, and action research. We will exclude duplicate publications, mixed-methods studies without the scope to separate qualitative data, and non-English or Chinese literature. Finally, the psychological adjustment communication system will be constructed initially based on a search in three aspects: helpfulness, affectivity, and affirmation.

### Phase 2: Qualitative study

In this phase, we will use semi-structured deep interviews to learn about the patient’s experience of psychological distress and the content of their needs in the helpful, emotional, and affirmative psychological support module and to understand the training content that parents need to provide emotional and psychological support to their children at a high risk of depression. This study will be conducted at the gynecology clinic of the Affiliated Hospital of Zunyi Medical University. The schedule, length, and place of the interview will be selected on the basis of participant preferences.

### Study sample

Participants will be selected through purposive sampling and selected for interviews according to the sampling criteria, and the interviews will be continued until data saturation. After no new topics are presented in the data analysis, two-sample sizes will be added to determine the final research sample size.

### Participants

The target population includes the general population of Guizhou Province in China who meet the following inclusion criteria: Chinese PCOS patients aged 10–19 years (28), Rotterdam diagnostic criteria recommended by the PCOS China Diagnosis and Treatment Guide in 2018 (29), self-reporting (with certain language comprehension and expression ability), and informed consent of subjects and family to participate in this study voluntarily. The exclusion criteria will be as follows: cognitive impairment and major mental disorders, other serious diseases of important organs, and other diseases that lead to elevated androgen levels and ovulation disorders.

### Data collection and analysis

The research team will consist of a PhD in nursing, a gynecologist carrying out research on PCOS, and two trained graduate students, all of whom will collaborate for data collection and analysis. The interviews with adolescents with PCOS will begin with the following questions:

“How did you feel during the treatment process?,” “What problems or difficulties did you encounter?,” “How did you solve these problems?,” “What are your needs?,” and “What support and assistance would you like to receive?”

The interviews with parents of the adolescents with PCOS will begin with these questions:

“Do you have problems communicating with your daughter in your daily life?,” “How did you cope with this?,” “What kind of guidance and help would you like to receive?,” “Would you be comfortable using a platform that provides guidance and assistance in communicating with your daughter through WeChat?,” and “What suggestions do you have for this platform?”

After each interview, the audio recordings were converted into written transcripts within 24 h, and the non-verbal information will be supplemented with interview notes to ensure accuracy and completeness and backed up to prevent loss. Using the Colaizzi content analysis method, two researchers will code, compare, corroborate, analyze, summarize, and distil themes. The theoretical module of the psychological adjustment communication system will be modified and improved based on the results of the qualitative research survey.

### Phase 3: The Delphi method

The Delphi method is intended to score and guide the importance and maneuverability of constructing psychological adjustment communication systems. This preliminary psychological adjustment communication system is composed of an expert letter questionnaire, which includes the research background, expert general situation questionnaire, and main body of the questionnaire. The expert will complete the questionnaire in accordance with the instructions on the form. Researchers will summarize, screen, and modify the opinions put forward by the experts on the psychological adjustment communication system, request the experts for consultation again, and repeat the process until the experts stop contributing opinions. Finally, the research group will discuss expert opinions and suggestions to further improve psychological adjustment communication.

### Participants

Expert selection criteria are as follows: medical experts, nursing experts, and psychologists in the field of gynecology, an experience at least 10 years to understand the contents of the letters; professional title is a senior professional title, bachelor’s degree or above, scientific research attitude, and the ability to provide more comprehensive opinions, and voluntarily participating in the research.

### Criteria and principles for revising indicators

#### Index revision criteria

Screening and revision according to expert scores, opinions, and suggestions. The inclusion principles of the index are as follows: the mean value of the importance assignment *X* > 3.5, and the coefficient of variation (CV) < 0.25. The principle of index modification is based on expert opinions and suggestions; corresponding modifications, additions, and deletions are carried out, and adjustments are made after repeated discussions by the research group.

### Construction of the psychological adjustment communication system

Finally, the research group will discuss expert opinions and suggestions to improve the construction of a psychological adjustment communication system. The psychological adjustment communication system for adolescents with PCOS at a high risk of depression will be developed on the basis of the results of expert consultation, and the psychological adjustment communication platform will be constructed through the combination of a WeChat official account and WeCom by computer software experts. The final system will consist of four main ports: the research team, parent, peer, and patient ports, each with different functional modules based on the role identity. The main function of these ports is for medical staff to provide disease consultation to patients through the official account, and parents to acquire some adolescent parent-child communication skills through the official account. Peer support volunteers will interact with patients through WeCom, etc. Patients, parents, medical staff, and peer support volunteers log in through the corresponding port to use the system.

### Phase 4: Quantitative study

During this phase, we shall utilize quantitative research methods with a before-after design to determine the usability and effectiveness of the psychological adjustment and communication platform for adolescents with PCOS who are at a high risk of depression.

### Study environment and population

This study will be conducted at the gynecology clinic of the Affiliated Hospital of Zunyi Medical University.

### Study sample

The target population for the study is adolescents with a confirmed diagnosis of PCOS based on the Rotterdam diagnostic criteria. In this study, the sample size has been calculated based on the study by Dana et al. (30): considering the weight variable, SD = 18, mean of paired differences = 11, α = 0.05, and power = 90%, the sample size was calculated using PASS15.0 software *N* = 31). Considering a loss of 20% of the sample, 39 participants will be included in this study.

### Inclusion criteria

#### Patients

Adolescents with PCOS at a high risk of depression who possess a smartphone, can skillfully operate the device, self-report (have a certain level of language understanding), and voluntarily participate in the study after providing informed consent will be included in the study.

#### Parents

Parents possessing a certain level of education, having no barriers to communication, owning a smartphone and being able to operate it, providing informed consent and volunteering to participate in the study, and having sufficient time to devote to training and intervention.

#### Peer support volunteers

Peer support volunteers with a certain level of education, a previous history of PCOS in adolescence, showing good control after treatment (normal hormone levels, normal ultrasound findings, and a normal depression score), possessing a smartphone, using it skillfully, volunteering to participate in this study, and having ample time and energy.

### Exclusion criteria

Patients, parents, and peer support volunteers who do not complete the intervention for any reason, such as cognitive impairment or severe mental illness, will be excluded from the study.

### Data collection methods

The baseline assessment will measure the psychological condition of adolescents with PCOS who are at a high risk of depression. The efficacy endpoint will then be measured at weeks 4, 8, and 12. A structured, validated, and reliable self-designed questionnaire will be used to collect data. On average, each respondent is expected to complete the questionnaire within 5 min. The instruments used are as follows:

#### Sociodemographic characteristics

Demographic characteristics will be measured using the personal information form developed by the researchers and consisting of questions pertaining to age, nationality, education level, living residence, years of PCOS, weight, height, body mass index, waist circumference, etc.

#### Child depression inventory

Depressive conditions will be measured using the Child Depression Inventory (CDI), which was translated into Chinese by David Yu (31). The validity and reliability of the Chinese version were assessed by Wu Wenfeng (32, 33). The Chinese version of the CDI contains five subscales: low self-esteem, negative affect, lack of pleasure, poor performance, and interpersonal problems. The scale has 27 items, each with three options for describing the level of depression, and is scored on a 0∼2 point scale, with higher scores indicating more severe depression. The scale can be applied to children and adolescents aged between 7 and 17 years. CDI has well-validated psychometric properties and has been used to reliably diagnose depression in medically ill populations. The total Cronbach’s alpha value was 0.85 in the reported study, and the test-retest reliability was 0.75 (34).

#### Medication compliance scale

Medication compliance in Chinese patients with chronic diseases has been measured using the Medication Compliance Scale (MCS) (35). This scale includes 16 items divided into two dimensions. Each item is scored on a 5-point scale with scores of 1–5. The higher the score, the better the patient’s adherence, with total scores < 51 indicating failing adherence, 51–67 indicating passing adherence, and ≥68 indicating good adherence. The total scale of career competencies showed excellent internal consistency, with a Cronbach’s α of 0.717.

#### Modified polycystic ovary syndrome health-related quality of life questionnaire

The Chinese version of the Modified Polycystic Ovary Syndrome Health-related Quality of Life Questionnaire (MPCOSQ) has been used to measure the health-related quality of life of PCOS patients in China (36). The MPCOSQ includes six dimensions covering 30 items. Each item was scored from 1 to 7, with 7 representing the ideal functional state and 1 representing the worst functional status. The lower the score, the higher was the patient’s attention to this dimension. The questionnaire assessed living conditions over the past 2 weeks. Cronbach’s α of the scale was 0.880, and the α-values of all dimensions were greater than 0.8.

#### Ascension insomnia scale

The sleep condition of the patients will be assessed using the Chinese version of Ascension Insomnia Scale (AIS) (37), which consists of eight questions rated on a 4-point Likert scale with scores of 0–3 ranging from “no problem” to “severe scenic impact.” A score of <4 was considered to indicate normal sleep, 4–6 indicated that sleep may be affected, and ≥6 indicated a sleep disorder. For internal consistency, Cronbach’s alpha was approximately 0.90, and the mean item-total correlation coefficient was approximately 0.70. For external validity, the correlations of the AIS-8 and AIS-5 scores with the Sleep Problems Scale score were 0.90 and 0.85, respectively (38).

### Data analysis

For participants undergoing the intervention, the data obtained at baseline and follow-up examinations at week 4, week 8, and week 12 and showing a normal or approximately normal distribution will be expressed as mean ± standard deviation (x ± s) and statistically analyzed by the paired-sample *t*-test, or analyzed by a Wilcoxon signed-rank sum test of paired samples.

## Discussion

The project focuses on adolescents with PCOS who are at a high risk of depression, which is an important population to study. Despite the increasing awareness among adolescents at a high risk of depression, medical staff still focus on psychotherapy, and preventive interventions for people at a high risk of depression are neglected (19, 20). Patients at a high risk for depression may eventually develop depression without intervention. Nevertheless, blind psychotherapy for this patient population will negatively influence the effectiveness of the therapy, exacerbate the psychological burden on the patient, and cause wastage of medical resources. Therefore, this study aimed to develop, implement, and evaluate a psychological adjustment communication system for adolescents with PCOS who are at a high risk of depression. We hope to offer strategies for preventive interventions in adolescents with PCOS who are at a high risk of depression.

The protocol is based on a theory of social support and involves designing a psychological adjustment communication system based on a literature review, qualitative studies, and the Delphi method. The proposed system aims to provide preventive psychological interventions for adolescents with PCOS at a high risk of depression and at different levels of social support, including help and affirmative support from medical staff, as well as emotional and affirmative support from parents and peers, with the aim of providing a fresh perspective on the existing psychological intervention methods.

This study will allow us to ascertain whether the psychological communication system can facilitate a positive psychological adjustment, enhance protective factors, promote stress management, decrease medical costs, reduce the prevalence of depression, improve health status, and increase the quality of life of adolescents with PCOS who are at a high risk of depression in China. The results of this before-after controlled trial will provide rigorous evidence for patients, healthcare providers, families, and other stakeholders on the feasibility and effectiveness of the psychological adjustment communication system in achieving outcomes that are meaningful for adolescents with PCOS at a high risk of depression and providing a better understanding and management of PCOS. Considering the potential clinical and cost-effectiveness of the strategies of this program, we sincerely hope that its success will help improve the quality of patients’ lives and their health status.

## Ethics statement

This study was reviewed and approved by the Medical Ethics Committee of Zunyi Medical University (2021) 1-093. Written informed consent was obtained from all participants/participants’ legal guardians/next of kin for their participation in this study.

## Author contributions

HT and LW have put the manuscript into writing. All authors contributed in the conceptualization of the model protocol with regard to the design and methods, as well as having read and commented on the manuscript text, and have approved of the final version of the manuscript.
